# Unconventional Deformation Behaviours of Nanoscaled High-Entropy Alloys

**DOI:** 10.3390/e20100778

**Published:** 2018-10-11

**Authors:** Yeqiang Bu, Shenyou Peng, Shiwei Wu, Yujie Wei, Gang Wang, Jiabin Liu, Hongtao Wang

**Affiliations:** 1 School of Materials Science and Engineering, Zhejiang University, Hangzhou 310027, China; 2 LNM, Institute of Mechanics, Chinese Academy of Sciences, Beijing 100190, China; 3 Laboratory for Microstructures, Shanghai University, Shanghai 200444, China; 4 Institute of Applied Mechanics, Zhejiang University, Hangzhou 310027, China

**Keywords:** nanoscaled high-entropy alloys, nanodisturbances, phase transformations, atomic-scale unstable

## Abstract

The bulk high-entropy alloys (HEAs) exhibit similar deformation behaviours as traditional metals. These bulk behaviours are likely an averaging of the behaviours exhibited at the nanoscale. Herein, in situ atomic-scale observation of deformation behaviours in nanoscaled CoCrCuFeNi face-centred cubic (FCC) HEA was performed. The deformation behaviours of this nanoscaled FCC HEA (i.e., nanodisturbances and phase transformations) were distinct from those of nanoscaled traditional FCC metals and corresponding bulk HEA. First-principles calculations revealed an obvious fluctuation of the stacking fault energy and stability difference at the atomic scale in the HEA. The stability difference was highlighted only in the nanoscaled HEA and induced unconventional deformation behaviours. Our work suggests that the nanoscaled HEA may provide more chances to discover the long-expected essential distinction between the HEAs and traditional metals.

## 1. Introduction

Traditional alloys, such as steels and copper alloys, are fabricated based on one or two principle constituent elements. Yeh et al. [1] proposed the concept of high-entropy alloys (HEAs) that provides a novel basis to design new alloys. These HEAs are composed of multi-principle elements at equiatomic or near-equiatomic ratios, distinguishing them from traditional alloys. Consequently, the deformation behaviours of HEAs are believed to be different from traditional alloys [2], but no convincing experiments have yet been reported to show an essential distinction of the plastic deformation behaviours between HEAs and traditional metals. On the contrary, most previous results in bulk HEAs have shown similar scenarios with traditional metals, in which the plastic deformation is primarily carried by dislocations or twins [3,4,5]. The underlying mechanisms of macroscopic mechanical responses are essentially the collective behaviours of atomic-scale configurations. Therefore, the physical processes during alloy deformation exhibit no remarkable features if the salient atomic configuration details are essentially blurred and only the average effect can be measured and observed [6,7]. As expected, such macroscopic deformation behaviours are generally controlled by only a few key parameters such as the elastic moduli, the stable and unstable stacking fault energies (SFEs), the microstructure parameters and the temperature.

Close observation at the nanoscaled regime where discrete plasticity dominates may uncover the essential features that distinguish the HEAs from traditional alloys. Extensive investigations using in situ high-resolution transmission electron microscopy (HRTEM) have revealed some interesting deformation behaviours of nanoscaled pure metals [8,9,10,11,12,13,14,15,16,17]. Size-dependent behaviours have thus been uncovered such as the reversible deformation twinning and detwinning processes found in nanoscaled W samples [12] and the dislocation-originated stacking fault tetrahedra in nanoscaled Au samples [13]. Meanwhile, surface-mediated plasticity deformation behaviours have frequently been observed such as the partial dislocations emitted from the surface in sub-10 nm-sized Au [14] and the liquid-like deformations in sub-10 nm-sized Ag nanoparticles [15]. It would be interesting to determine whether nanoscaled HEAs behave similarly in the discrete plasticity regime, in contrast to their collective behaviour. The face-centred cubic (FCC) HEA CoCrCuFeNi is a typical HEA which was proposed in the earliest paper about the concept of HEA [1]. Numerous research papers related to HEAs were applied in CoCrCuFeNi from then. Therefore, CoCrCuFeNi was taken as a model HEA to reveal the obscured potential high-entropy effect on plastic deformation behaviours at the nanoscale in this work.

## 2. Materials and Methods

The CoCrCuFeNi button ingots were prepared by melting high-purity Co, Cr, Cu, Fe and Ni at equiatomic ratios in a vacuum arc furnace. During specimen preparation, the ingot was first sliced into a rod of 0.25 × 0.25 × 10 mm^3^ in dimension. Firstly, we employed a tungsten carbide cutter to make a pair of gaps on the surface of the rod. Then, we used a plier to pull this rod apart in the long dimension and then led to the formation of fresh triangular nano-tips on the fractured surface, which served as the specimens for the in situ TEM experiments (Figure S1 in supplementary). A JEM-2100F field emission TEM equipped with a Nanofactory TEM-scanning tunnelling microscope (STM) sample holder was used in the in situ TEM experiments. Two fractured ends exhibiting nano-tips were mounted on the sample holder, with one at the fixed end of the holder and the other at the piezo-manipulator end (Figure S2 in Supplementary Materials). One nano-tip could thus be driven to touch the other nano-tip on the counter fractured surface, guided by the piezo-actuated nanoscaled manipulator. A nanoscaled welding joint 10–30 nm in size could then be formed instantly via pulsed joule heating. Uniaxial tensile stress could be applied by step-by-step retraction via the nano-manipulator. This method essentially furnished the fabrication, mechanical testing and easily atomic-resolution observation of the nanoscaled HEA. The structure of the nanoscaled HEA fabricated by this method is shown in Figure S3. The crystalline interplanar spacing of the $\left(\bar{1}1\bar{1}\right)$ plane is 2.07 Å in the obtained nanoscaled HEA, i.e., its lattice parameter is 3.59 Å. This is consistent with the reported lattice parameter (3.579 Å) of the bulk CoCrCuFeNi [1,18]. This shows that the structure of the nanoscaled HEA is not affected by the process of preparation. Meanwhile, there is no oxide layer on the surface of the nano-tips before the fabrication of the nanoscaled samples, as shown in Figure S4, which indicates that there is no impact of the oxide layer on the deformation behaviours (These nano-tips were exposed to air for only a few minutes.). The energy dispersion spectrum (EDS) mapping results are shown in Figure S5, which indicates that the constituent elements distribute uniformly in the nao-tip (EDS hardly displays the details of composition in several-atoms scale, even the atomic resolution EDS only gives the statistical chemical composition of each atom column in planar view).

The first-principles calculations were performed using the Vienna ab initio simulation package based on density functional theory, wherein all of the HEA samples were relaxed to the energy precision of 0.01 meV. The SFE was calculated with *E*_sf_ = (*E*_fault_ − *E*_perfect_)/*A*, where *E*_perfect_ and *E*_fault_ are the free energy of perfect and faulted structures, respectively; and *A* is the area of each layer. The structure with one layer fault was obtained by a rigid displacement between two adjacent layers, where the magnitude was equal to that of the Burgers vector, ***b_p_*** = 1/6<112>.

## 3. Results

### 3.1. Nanodisturbances

**Figure 1:**
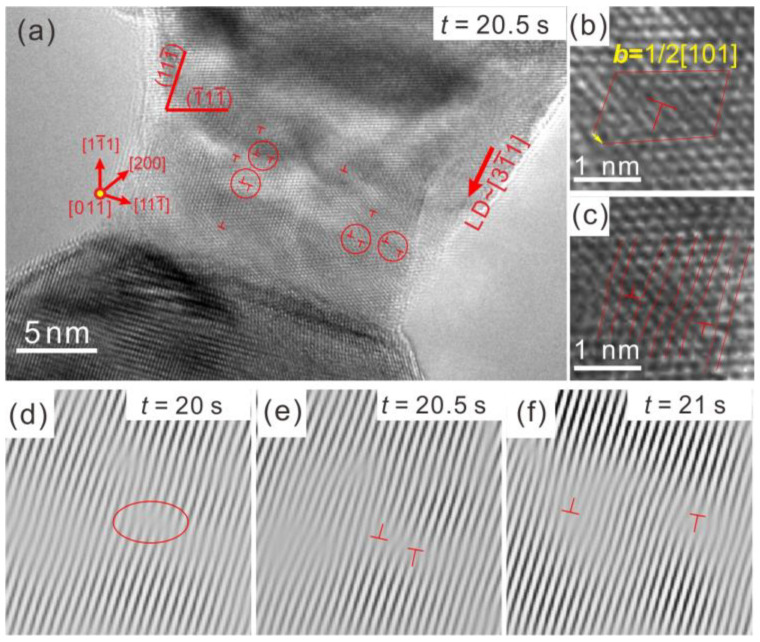
The nanodisturbances in the nanoscaled high-entropy alloy (HEA). (**a**) deformed nanoscaled HEA sample containing several dislocations (Beam // [011], loading direction (LD) ≈ $\left[3\bar{1}1\right]$); (**b**) analysis of the Burgers vector; (**c**) enlarged image of the dislocation dipole; (**d**–**f**) Inverse fast Fourier transform (IFFT) images of the one-dimensional {111} plane fringes showing the formation and expansion of the dislocation dipole.

Figure 1a displays a nanoscaled HEA during in situ straining in the TEM (Vedio S1 in Supplementary Materials), where the loading direction (LD) is around $\left[3\bar{1}1\right]$ and the beam zone is [011]. The deformed nanoscaled HEA contains several dislocations. The deformation of this nanoscaled HEA is closely related to the behaviours of dislocations. Dislocation cores are labelled in Figure 1 by “┴”, and an enlarged image of a dislocation core is shown in Figure 1b. According to the analysis of the Burgers circuit, these are full dislocations with ***b*** = 1/2[101]. Some of the dislocations (circled in Figure 1a) appear in pairs and are thus dislocation dipoles, seen in an enlarged image in Figure 1c. Using in situ HRTEM, we could dynamically observe the evolution of these dislocation dipoles. Figure 1d–f show inverse fast Fourier transform (IFFT) images of the one-dimensional {111} plane fringes in the area around a dislocation dipole. At *t* = 20 s, the circled area exhibited a distorted lattice which was induced by high stress in the nanoscaled HEA. At *t* = 20.5 s, a dislocation dipole pair was produced in the distorted area. Under stress driving, the dislocation dipole was observed to expand along the $\left(\bar{1}1\bar{1}\right)$ slip plane at *t* = 21 s. This deformation mode could be called nanodisturbance, which has been proposed on body-centred cubic (BCC) “gum metal” by Gutkin et al. [19]. This kind of dislocation dipole nucleation and expansion could act as a mechanism of dislocation multiplication during the deformation process of the nanoscaled HEA.

### 3.2. Phase Transformation

**Figure 2:**
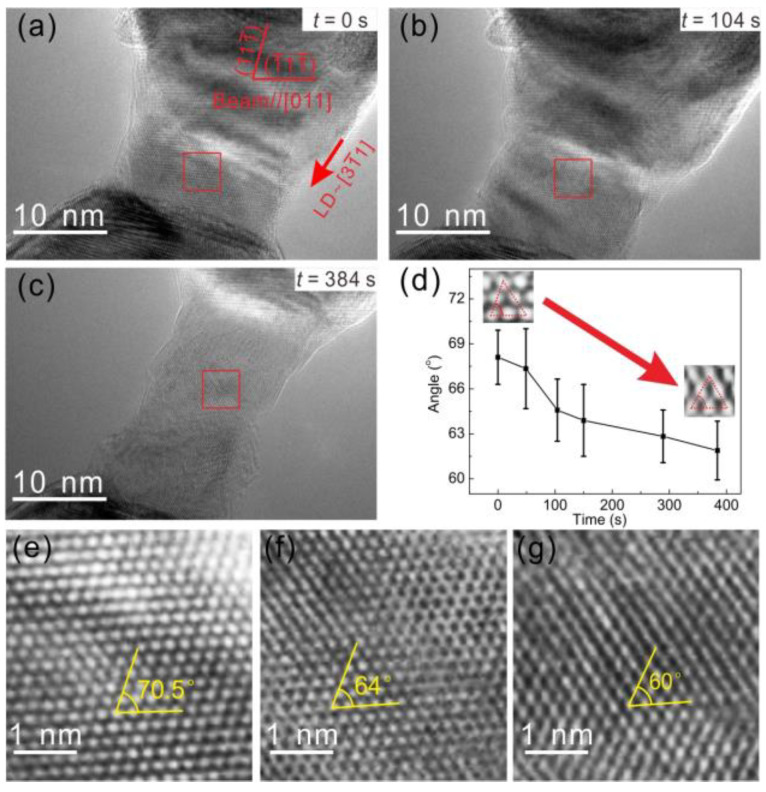
The phase transformation from face-centred cubic (FCC) to body-centred cubic (BCC) in the nanoscaled HEA. (**a**–**c**) elongation process of the nanoscaled sample (Beam // [011], LD ≈ $\left[3\bar{1}1\right]$); (**d**) variation of the angle between two close-packed planes in the red square area during in situ tension; (**e**–**g**) High-resolution transmission electron microscopy (HRTEM) images of the red square zones in (**a**–**c**), respectively.

Figure 2a–c shows the tensile process of a nanoscaled HEA sample (Video S2 in Supplementary Materials), where the LD is around the $\left[3\bar{1}1\right]$ and beam zone is [011]. Figure 2e–g show enlarged images corresponding to the areas in red squares in Figure 2a–c, respectively. As shown in Figure 2e, the crystal lattice exhibits clear characteristics of a FCC structure; i.e., the angle between two close-packed planes in the {111} family is 70.5°. As the nanoscaled HEA continues to be stretched, the angles between two {111} planes reduce to 64° at *t* = 104 s (Figure 2f), and then to 60° at *t* = 384 s (Figure 2g). The angle of 64° indicates that the lattice structure deviates from the original FCC structure notably. The angle of 60° represents a typical BCC structure with a [111] zone. Figure 2d plots the angles between the close-packed planes in the red square areas of Figure 2a–c as a function of time under stress, where the angle exhibits a slow and successive transition from ~70° to ~60°. The change of the angle corresponds to the transition that the initial FCC lattice transforms to BCC lattice during in situ tension. The resultant orientation relationship between the FCC and BCC agrees with the K–S relationship, i.e., [011]_FCC_ // [111]_BCC_, $\left(\bar{1}1\bar{1}\right)$_FCC_ // $\left(\bar{1}10\right)$_BCC_. The transformation process could be explained by K–S model. The FCC lattice sheared 19.5° along the <112>_FCC_ direction on the {111}_FCC_ plane and sheared 10.5° along the <110>_FCC_ direction on the {112}_FCC_ plane. Therefore, the stress-induced FCC→BCC transition realized and the BCC lattice formed. The slow and successive transformation was believed to relate with the high lattice friction in HEAs [20,21,22], the transformation dislocations slip and lattice shear more slowly in HEAs compared to the traditional abrupt Martensitic transformation. Such stress-induced FCC→BCC transition has a good reproducibility, and Figure S6 shows another example.

### 3.3. Fluctuation of Stacking Fault Energy

**Figure 3:**
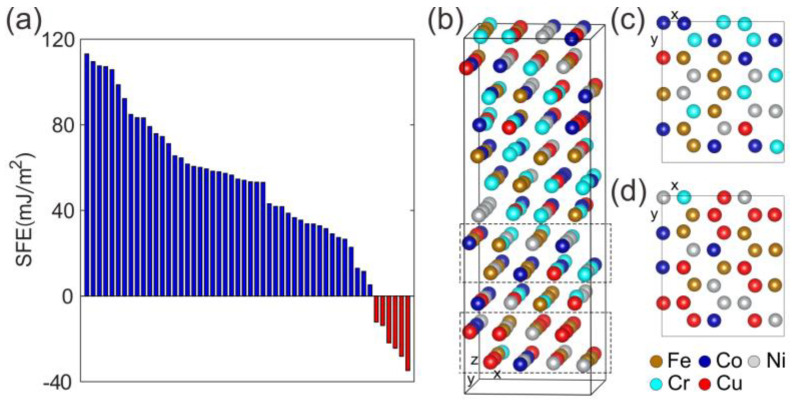
The fluctuant distribution of the stacking fault energies (SFEs). (**a**) the SFEs of 52 independent calculations; (**b**) typical structure of a calculation sample; (**c**) atomic configuration producing a negative SFE of −24 mJ/m^2^ from the upper dashed box in (**b**); (**d**) atomic configuration producing a high SFE of 109 mJ/m^2^ from the lower dashed box in (**b**).

The SFE is one of the most significant parameters determining the deformation behaviours of alloys, and is closely related to phase transformation and structural stability. Herein, we employed first-principles calculations to determine the SFE of this HEA, and the results of 52 independent SFE calculations are shown in Figure 3a. These results exhibit a fluctuant distribution of the SFE in HEA, where the SFE value covers a wide range and even is negative. The negative SFE values indicate that some of the HEA atomic configurations are unstable. The instability seems to be strongly correlated with the non-uniform distribution of atoms. As an example, we analyze a typical calculation sample illustrated in Figure 3b and find that the atoms in the dashed boxes (also seen in Figure 3c,d) are not a uniform distribution of all kinds of elements in CoCrFeNiCu. Some areas have more Co and Cr atoms and less Cu atoms (Figure 3c), which leads to a negative SFE of −24 mJ/m^2^. Some areas have more Cu atoms and less Co and Cr atoms (Figure 3d), which leads to a SFE as high as 109 mJ/m^2^. We conclude that the elemental inhomogeneity at the atomic scale leads to SFE difference in local, and the fluctuant distribution of the SFE induces a stability difference at atomic-scale. However, such fluctuant SFE is averaged in the bulk HEAs, the atomic-scale stability difference is also blurred. The small-scaled sample size may highlight such atomic details.

## 4. Discussion

In such in situ HRTEM experiments, the effects of electron beam on the nanoscaled samples should be verified and eliminated. Although the CoCrCuFeNi HEA was verified to be stable under the severe electron irradiation [23], all in situ experiments are still performed under the weak electron beam for minimizing the effect of electron irradiation. Meanwhile, our verification experiments (see Supplementary Figure S6) and theoretical estimation (see Appendix A) confirm the negligible influence of electron irradiation on the in situ experiments.

Nanodisturbances could be an effective mechanism for dislocation multiplication, and the process of nanodisturbances evolving into dislocation dipoles has been observed in the BCC gum metal [19,24]. However, there has not yet been any experimental observation of this novel dislocation-generating mechanism for FCC structure metals. Some theoretical calculations have investigated the nanodisturbance phenomenon in Au and Cu nanowires [25,26], indicating that the nanodisturbance deformation mode could dominate over traditional dislocation generation at high stresses and 0 K. Obviously, the temperature condition of 0 K was not satisfied in this work. However, we still observed the nanodisturbance deformation mode in FCC HEAs for the first time. The high-level stress in nanoscaled samples and the intrinsic features of HEAs both play significant roles on the occurrence of the nanodisturbances. On the one hand, we observed nanodisturbance in the nanoscaled FCC HEA at the relative loose condition (at room tempreture). On the other hand, nanodisturbance is hardly observed in the bulk HEAs because of the relative low stress level in the bulk HEA. Furthermore, we believe that it is the nanoscaled size that triggers the emergence of the intrinsic features of the HEA.

The previous works showed that there are no phase transformation during the deformation process [27]. However, herein we observed the FCC→BCC transformation in nanoscaled CoCrCuFeNi HEA. A similar transformation from FCC to body-centered tetragonal (BCT) has been observed in nanoscaled fractured Au [14,28], where the phase transformation therein was considered to be stimulated by the relaxation of surface stress. The surface stress has an inverse relationship with the sample size, and thus surface stress of a nanoscaled sample is sufficiently high to stimulate a transformation. However, in this study, the phase transformation in the nanoscaled HEA occurred before fracturing, and thus the surface stress had not been completely released. Therefore, the cause of phase transformation in the nanoscaled HEA is not solely surface stress, but an important role may also be played by the unstable nature of nanoscaled HEA. The combined effect of high surface stress and an unstable nature, therefore, stimulates the occurrence of the phase transformation.

Both nanodisturbances and phase transformations are not regular deformation behaviours in bulk CoCrCuFeNi HEA. These unconventional deformation behaviours observed in nanoscaled HEA are believed to be related to atomic configuration details present at such a small scale. Our first-principles calculations could well illuminate the unconventional deformation behaviours in such nanscaled HEAs. The first-principles results show that the elemental inhomogeneity at the atomic scale leads to SFE difference in local, and the fluctuant distribution of the SFE induces a stability difference of atomic-scale HEA. However, the entire structure averaged in the bulk HEA possesses a constant SFE. The stability difference can be outlined and plays a dominant role only when the sample dimensions reach the nanoscale. At that time, nanoscaled HEA exhibits deformation behaviours different with bulk counterparts. We further speculate that the nanoscaled HEA provides more of a chance to discover the long-expected essential distinction between the HEAs and traditional metals.

Besides the FCC HEAs, the BCC and hexagonal close-packed (HCP) HEAs may also possess some distinct characteristic physical properties but are blurred in bulk. It is worth investigating the other nanoscaled HEAs with various structures in future research to reveal the essential distinction between the HEAs and traditional metals.

## 5. Conclusions

In summary, we employed in situ HRTEM to investigate the deformation behaviours of nanoscaled HEA. Unconventional deformation behaviours (i.e., nanodisturbances and phase transformations) were observed in the nanoscaled HEA. The first-principles calculations revealed obvious fluctuant distribution of the SFE at atomic scale, which was resulted from the elemental inhomogeneity. The SFE fluctuation leaded to stability difference at the atomic scale, which plays a dominant role in the deformation of the nanoscale sample but tiny roles in bulk counterparts. The nanoscaled HEA provided a chance to highlight the stability difference and therefore exhibited unconventional deformation behaviours. Our investigations reveal some HEA features and are significant for understanding the nature of HEA.

## Supplementary Materials

The following are available online at http://www.mdpi.com/1099-4300/20/10/778/s1, Figure S1: The nano-tips on the fractured surface, Figure S2: Two teared parts with nano-tips are mounted on a Nanofactory transmission electron microscope (TEM)-scanning tunnelling microscope (STM) TEM holder, Figure S3: The structure keep unchanged during the preparation process, Figure S4: The high-resolution TEM image of the nano-tip, Figure S5: The verification experiments about the effects of electron irradiation, Video S1: Nanodisturbances deformation mode in the nanoscaled HEA, Video S2: Phase transformation in the nanoscaled HEA.

## Appendix A. Theoretical Estimation of the Effects of Electron Irradiation

The effects of electron irradiation could be divided into two aspects, i.e., knock-on displacement and heating effect. We could not observe any surface diffusion or atoms hopping occurs in the absence of external force. Therefore, the knock-on displacement driven by electron irradiation has no influence on the nanoscaled samples. For the heating effect, the estimation was based on the Fisher’s model [29] and the Bethe–Bloch equation [30]. Herein, the nanoscaled samples are all with a certain thickness (~10 nm), and exposed at the weak beam intensity. Fisher has proposed a model to estimate the temperature increase induced by electron irradiation [29]:

(1) $$\Delta T=\frac{I}{4\pi Ke}(\frac{\Delta E}{d})(1+2\ln\frac{b}{r_{0}}),$$

where ∆*T* is the maximum temperature rise heated by electron irradiation, *K* is the thermal conductivity of the sample, *I* is the beam current, ∆*E* is the total energy loss per electron in a sample of thickness *d*, *b* is the radius of the heat sink, and *r_0_* is the beam radius. The term $\frac{\Delta E}{d}$ could be approximately equal to $\frac{dE}{dx}$ (where *x* is the position in thickness), which could be calculated by the Bethe–Bloch equation [30], as follows:

(2) $$-\frac{dE}{dx}=\frac{2\pi Z\rho {\left(e^{2}/4\pi \varepsilon_{0}\right)}^{2}}{m\nu^{2}}\left\{\ln\left[\frac{E{\left(E+mc^{2}\right)}^{2}\beta^{2}}{2I_{e}^{2}mc^{2}}\right]+(1-\beta^{2})-(1-\sqrt{1-\beta^{2}}+\beta^{2})\ln2+\frac{1}{8}{\left(1-\sqrt{1-\beta^{2}}\right)}^{2}\right\},$$

where *Z* is the atomic number of the samples, *ρ* is the atomic density, $\varepsilon_{0}$ is the vacuum dielectric constant, *m* is the electron rest mass, *ν* is the electron velocity, *c* is the speed of light, *E* is the electron energy, *I_e_* is the average excitation energy of electrons in the target, and *β* = *ν*/*c*.

We take the *Z*_ave_ = (*Z*_Co_+ *Z*_Cr_+ *Z*_Cu_+ *Z*_Fe_+ *Z*_Ni_)/5 = 26.8 as the atomic number of this HEA samples containing Co, Cr, Cu, Fe and Ni five principle elements. *ρ* = *ρ*_mass_/((*m*_Co/atom_+ *m*_Cr/atom_+ *m*_Cu/atom_ + *m*_Fe/atom_+ *m*_Ni/atom_)/5) = 7.231 × 1027 m^−3^. The thermal conductivity of this HEA is estimated to be *K*= 16.2 W·m^−1^·K^−1^. Given *I* = 4.8 nA, *e* = 1.6 × 10^−19^ C, *b* = 1.5 mm, *r*_0_ = 200 nm, $\varepsilon_{0}$ = 8.85 × 10^−12^ F·m^−1^, *m* = 9.3 × 10^−31^ kg, *ν* = 2.0837×10^8^ m·s^−1^, *c* = 3.0 ×10^8^ m·s^−1^, *E* = 200 KeV, *I_e_* = 8.8*Z* = 235.8 eV, the estimated results of the maximum temperature increase of the HEA sample is 0.061 K. Therefore, the heating effect induced by electron irradiation could be neglected.
