# Supplementary material for: Unconventional Deformation Behaviours of Nanoscaled High-Entropy Alloys
Source: Entropy (Basel). 2018 Oct 11;20(10):778. doi: 10.3390/e20100778 (PMC7512340; doi:10.3390/e20100778)
Supplement: Supplementary file 1 [file entropy-20-00778-s001.zip › entropy-361604-SI.pdf]

Supplementary Materials: Unconventional Deformation Behaviours of Nanoscaled High-entropy Alloys

Yeqiang Bu, Shenyou Peng, Shiwei Wu, Yujie Wei, Gang Wang, Jiabin Liu and Hongtao Wang

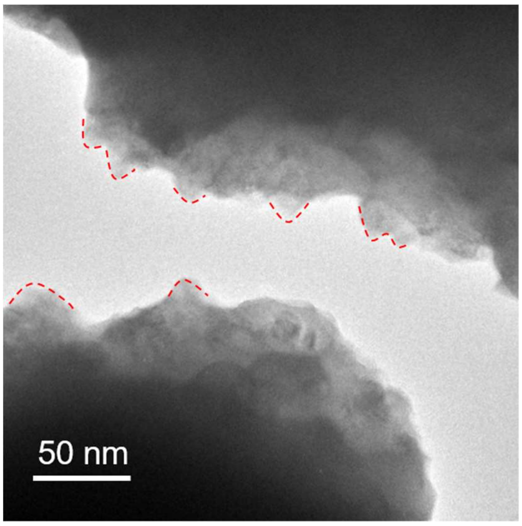

Figure S1. The nano-tips on the fractured surface.

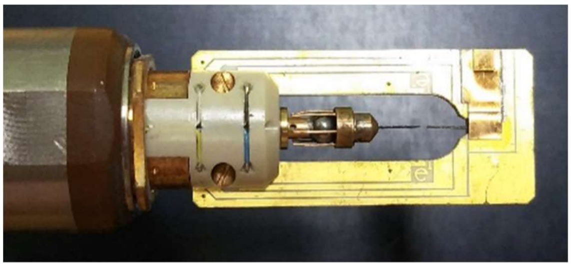

Figure S2. Two teared parts with nano-tips are mounted on a Nanofactory transmission electron microscope (TEM)-scanning tunnelling microscope (STM) TEM holder.

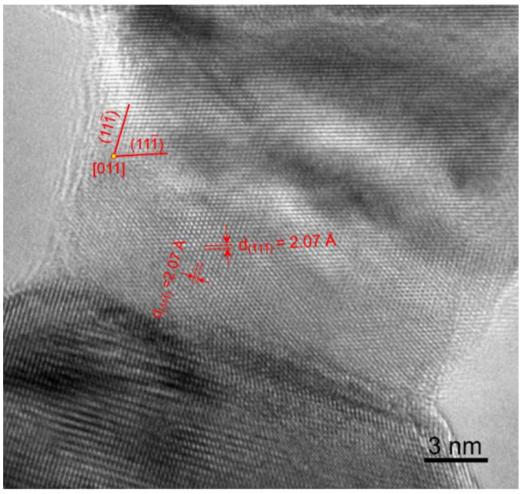

Figure S3. The structure keep unchanged during the preparation process. The crystalline interplanar spacing of the $(\bar{1}1\bar{1})$ plane and $(11\bar{1})$ are 2.07 Å in the nanoscaled HEA, *i.e.* its lattice parameter is 3.59 Å.

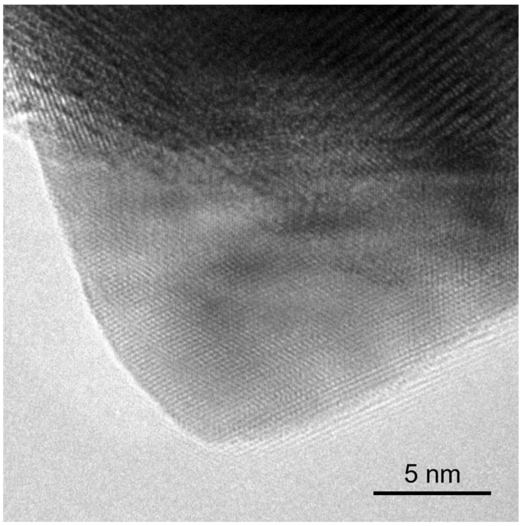

Figure S4. The HRTEM image of the nano-tip. There are no oxide layer on its surface.

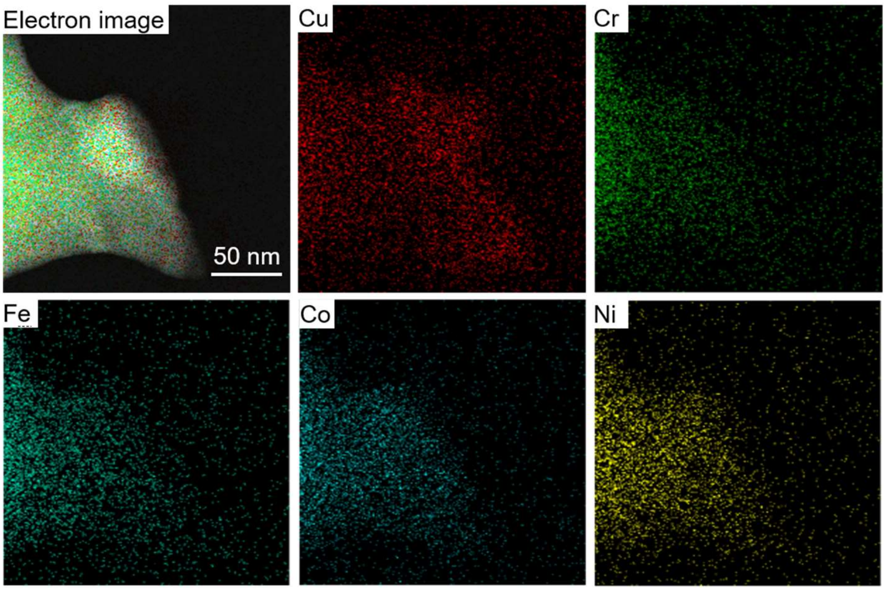

Fugure S5. The EDS mapping of the nano-tip shows the elements uniformly distribute in the nano-tip.

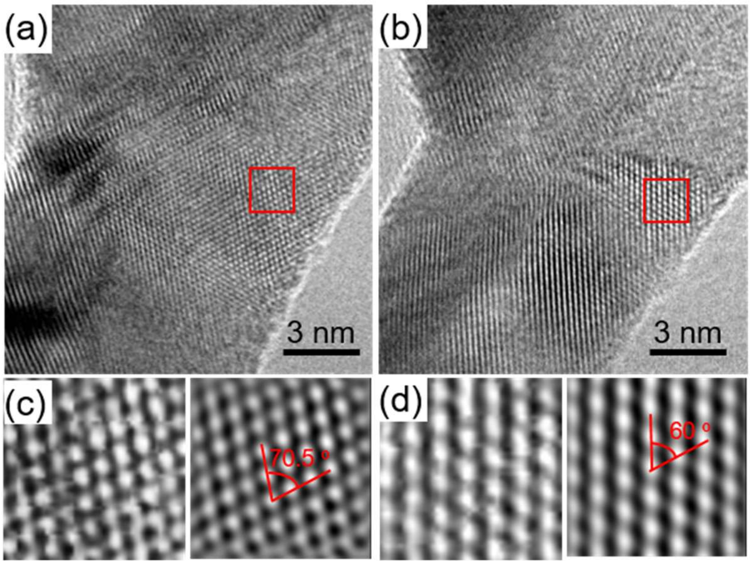

Figure. S6 The another example of the phase transformation. (a) The nanoscaled HEA sample with FCC structure; (b) the nanoscaled HEA after pulling; (c) is enlarged image and inverse fast Fourier transform image corresponding to the red square area in (a), and its angle between two close package plane is 70.5 °, which is the typical characteristic of FCC lattice; (d) is enlarged image and inverse fast Fourier transform image corresponding to the red square area in (b), and its angle between two close package plane is 60 °, which is the typical characteristic of BCC lattice.

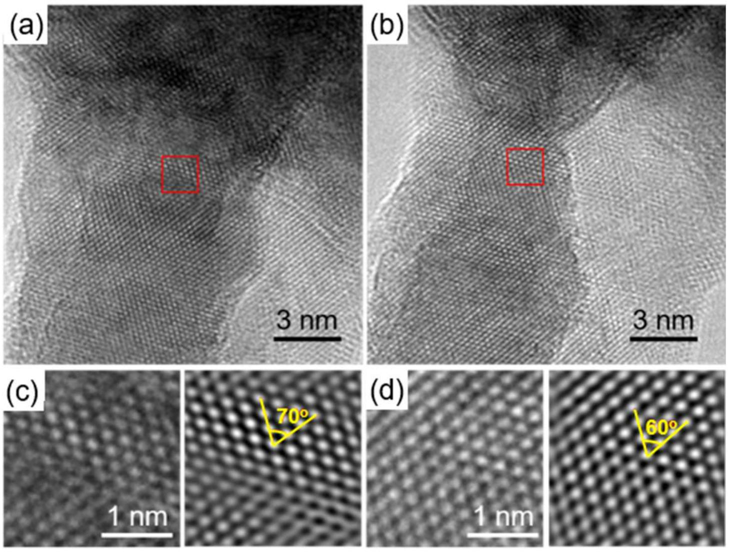

Figure S7. The verification experiments about the effects of electron irradiation. (**a**) The nanoscaled HEA sample with FCC structure, and we pulled the sample at beam-blank condition; (**b**) the nanoscaled HEA after pulling at beam-blank condition; (**c**) is enlarged image and inverse fast Fourier transform image corresponding to the red square area in (**a**), and its angle between two close package plane is 70 °, which is the typical characteristic of FCC lattice; (**d**) is enlarged image and inverse fast Fourier transform image corresponding to the red square area in (**b**), and its angle between two close package plane is 60 °, which is the typical characteristic of BCC lattice. The phase transformation was still observed at beam-blank condition, and confirm the negligible effect of the electron irradiation.
